# Dexamethasone Implant in Epiretinal Membrane Surgery: A Clinically Oriented Narrative Review of Current Evidence, Biomarkers and Patient Selection

**DOI:** 10.3390/jcm15155966

**Published:** 2026-07-30

**Authors:** Clemente Maria Iodice, Michele Cillis, Danilo Iannetta, Rabia Bourkiza, Michele Reibaldi, Paola Marolo, Enrico Borrelli, Giulia Midena, Georgios D. Panos, Mariantonia Ferrara, Josef Guber, Aman Chandra, Roger Wong, Theo Empeslidis, Lorenzo Motta

**Affiliations:** 1 Multidisciplinary Department of Medical, Surgical and Dental Sciences, Eye Clinic, University of Campania Luigi Vanvitelli, 80131 Naples, Italy; 2 Ophthalmology Unit, Department of Neuroscience, University of Padua, 35122 Padua, Italy; lorenzo.motta@unipd.it; 3 Department of Sense Organs, Sapienza University of Rome, 00185 Rome, Italy; iannettadanilo@gmail.com; 4 Ophthalmology Department, Central Middlesex Hospital, London Northwest University Healthcare NHS Trust, London HA1 3UJ, UK; 5 Section of Ophthalmology, Department of Surgical Sciences, University of Turin, 10124 Turin, Italy; michele.reibaldi@unito.it (M.R.); paola.marolo@unito.it (P.M.); enrico.borrelli@unito.it (E.B.); 6 IRCCS—Fondazione Bietti, 00184 Rome, Italy; giulia.midena@fondazionebietti.it; 7 First Department of Ophthalmology, AHEPA University Hospital, School of Medicine, Aristotle University of Thessaloniki, 541 24 Thessaloniki, Greece; gdpanos@gmail.com; 8 Division of Ophthalmology and Visual Sciences, School of Medicine, University of Nottingham, Nottingham NG7 2RD, UK; 9 Department of Medical and Surgical Specialties, Radiological Sciences, and Public Health, University of Brescia, 25121 Brescia, Italy; mariantonia.ferrara@gmail.com; 10 Augenklinik Thurgau, 8500 Frauenfeld, Switzerland; josef.guber@augenchirurgie.ch; 11 Department of Ophthalmology, Southend University Hospital, Mid and South Essex NHS Foundation Trust, Southend-on-Sea SS0 0RY, UK; amanchandra@gmail.com; 12 Ophthalmology Department, St Thomas’ Hospital, London SE1 7EH, UK; roger.wong@gstt.nhs.uk; 13 Stoneygate Eye Hospital, Leicester LE2 2PN, UK; theo@empeslidis.com

**Keywords:** epiretinal membrane, pars plana vitrectomy, dexamethasone intravitreal implant, cystoid macular edema, macular surgery

## Abstract

**Background:** Epiretinal membrane (ERM) is a fibrocellular preretinal proliferation composed of fibroblasts, glial cells, and hyalocytes overlying the internal limiting membrane. Pars plana vitrectomy (PPV) with membrane peeling is the standard surgical treatment, but postoperative cystoid macular edema (CME) can limit visual recovery. Inflammation plays a key role in CME pathogenesis, and corticosteroids may help reduce the inflammatory response associated with both ERM-related traction and surgical trauma. Therefore, sustained-release dexamethasone (DEX) intravitreal implants have been investigated as an adjunct to surgery. **Methods:** This SANRA-guided narrative review evaluated studies investigating DEX implantation during PPV for ERM surgery or as treatment for persistent postoperative CME. Eligible studies included human clinical studies reporting functional, anatomical, biomarker, or safety outcomes following DEX use in the ERM surgical setting. **Results**: DEX implantation appears to promote faster resolution of postoperative inflammation and CME, with consistent improvements in anatomical parameters such as central macular thickness. However, the impact on visual acuity remains variable, with some studies reporting earlier functional recovery and others showing no significant long-term benefit. Reported adverse events mainly include intraocular pressure elevation manageable with medical therapy and cataract progression. Available evidence suggests that intraoperative DEX implantation is associated with more consistent anatomical benefit than superior long-term functional improvement, while delayed DEX administration may benefit selected eyes with persistent postoperative cystoid macular edema. **Conclusions**: Current evidence suggests that DEX implantation may represent a potentially useful adjunctive strategy in selected patients undergoing ERM surgery, particularly in eyes at higher risk of postoperative inflammation or cystoid macular edema. While available studies suggest potential anatomical benefits, evidence regarding superior long-term functional outcomes remains inconsistent. Moreover, while current evidence suggests that DEX implantation may accelerate anatomical recovery and improve postoperative inflammatory control in selected patients, long-term functional superiority over PPV alone, including sustained BCVA improvement and patient-reported visual outcomes such as metamorphopsia, remains unproven. Larger prospective randomized studies are needed to better define optimal patient selection and treatment timing.

## 1. Introduction

Epiretinal membrane (ERM) is a common retinal condition caused by cellular proliferation and metaplasia leading to the formation of a pathological fibrocellular layer overlying the retina [1]. The surgical excision by means of pars plana vitrectomy (PPV) with ERM peeling represents the only therapeutic option available for symptomatic ERMs aimed at improving the traction-related vision changes [2].

However, despite several studies describing significant anatomical and functional improvements after surgical ERM removal, postoperative cystoid macular edema (CME) remains a limiting factor to the visual outcome, with a reported incidence that ranges from 10% to 40% [3].

Intravitreal steroids, including dexamethasone (DEX) and triamcinolone, have been used with similar modalities to treat postoperative CME after ERM peeling [4]. Compared with topical anti-inflammatory therapy, systemic corticosteroids, or repeated intravitreal injections, sustained-release DEX implantation offers the potential advantage of prolonged intraocular drug delivery, more consistent postoperative inflammatory control, and reduced treatment burden in selected patients. In particular, the slow-releasing DEX intravitreal implant has been proposed as the key steroid in accelerating the resolution of postoperative inflammation, leading to faster and better outcomes after surgery [3,4].

However, the efficacy, safety, and optimal role of DEX implantation in ERM surgery remain debated. Although several studies and evidence syntheses have evaluated DEX use in this setting, important clinical questions remain unresolved, including optimal timing of administration, patient selection, biomarker-guided risk stratification, and long-term risk–benefit balance. The central clinical question remains whether adjunctive DEX implantation provides sufficient functional and anatomical benefit over PPV alone to justify its additional risks and costs, and which patient subgroups are most likely to benefit from this strategy. This narrative review critically evaluates the current evidence on DEX use in ERM surgery, with particular emphasis on clinical outcomes, imaging biomarkers, patient selection, and future therapeutic perspectives.

## 2. Methods

This narrative review was conducted in alignment with the Scale for the Assessment of Narrative Review Articles (SANRA) recommendations to improve methodological rigor, transparency, and reporting quality.

A structured literature search was conducted using PubMed and Embase databases from inception to 31 January 2025. Search terms included combinations of “dexamethasone implant”, “Ozurdex”, “intravitreal steroid”, “pars plana vitrectomy”, “vitrectomy”, “epiretinal membrane”, “membrane peeling”, “cystoid macular edema”, and “postoperative macular edema”, combined using Boolean operators (AND/OR) to identify studies relevant to DEX implantation in ERM surgery and postoperative CME. Only studies published in English and involving human subjects were considered. Eligible studies included prospective and retrospective clinical studies evaluating DEX implantation either in conjunction with PPV and ERM peeling or for the treatment of persistent postoperative CME following ERM surgery. Relevant case series and selected case reports were also considered when they provided clinically meaningful information regarding efficacy or safety. Studies not specifically addressing ERM surgery, non-clinical studies, conference abstracts, editorials, and publications lacking original clinical data were excluded.

The retrieved articles were screened based on title and abstract, followed by full-text assessment of potentially relevant studies. Reference lists of included articles were also manually reviewed to identify additional relevant studies.

For structured evidence synthesis, studies were categorized a priori into two main clinical subgroups: (1) studies evaluating intraoperative or perioperative DEX implantation in conjunction with PPV and ERM peeling, and (2) studies evaluating delayed DEX administration for persistent postoperative cystoid macular edema. These groups were analyzed separately because they represent distinct clinical scenarios with different therapeutic objectives and outcome expectations.

Given the narrative design of this review and the heterogeneity of included studies in terms of study design, DEX timing, outcome measures, and follow-up duration, no formal quantitative synthesis was performed. The methodological quality of included studies was assessed qualitatively using a domain-based approach focusing on study design, sample size, control group availability, selection bias, confounding risk, timing of DEX administration, outcome reporting, and safety reporting.

## 3. Mechanism of Action and Pharmacokinetics of Dexamethasone Implants

DEX is a potent corticosteroid with anti-inflammatory and anti-edematous properties. It is thought to exert its therapeutic effects through modulation of multiple inflammatory pathways, including suppression of pro-inflammatory cytokine release, reduction of vascular permeability, stabilization of the blood–retinal barrier, and attenuation of inflammatory cell recruitment. These mechanisms may contribute to reduced retinal edema and improved postoperative anatomical recovery [3].

In particular, the injection of a sustained-release intravitreal preparation has been reported to be beneficial in the treatment of retinal conditions. Many of the pathologies treated by vitreoretinal surgery can trigger pro-inflammatory responses and CME. Retinal surgery itself can further amplify these inflammatory reactions, potentially worsening or facilitating the onset of CME. Also, ERMs may be primarily associated with CME as a result of traction on local vascular structures.

The key feature of the intravitreal, slow-release DEX implant (Ozurdex^®^, Allergan, Inc., Irvine, CA, USA) is the formulation of the biodegradable polylactic-co-glycolic acid copolymer that directly releases the active ingredients within the vitreous cavity, before completely dissolving [5,6]. DEX implant was reported to maintain high concentrations in the vitreous cavity for up to 60 days before declining over 6 months after the injection. The implant ultimately dissolves completely within the vitreous cavity [6].

## 4. Inflammatory Background of ERM Relevant to Postoperative CME

Although ERM has traditionally been regarded as a predominantly mechanical disorder characterized by tangential traction at the vitreoretinal interface, increasing evidence suggests that inflammatory mechanisms also contribute significantly to both disease progression and postoperative outcomes [7,8,9]. ERM formation is now understood as a multifactorial process involving glial proliferation, Müller cell activation, microglial recruitment, and extracellular matrix remodeling, ultimately generating a chronic low-grade inflammatory retinal microenvironment [7,8,9].

Several inflammatory mediators have been implicated in ERM pathophysiology and postoperative cystoid macular edema (CME), including interleukin-6 (IL-6), vascular endothelial growth factor (VEGF), monocyte chemoattractant protein-1 (MCP-1), tumor necrosis factor-alpha (TNF-α), and transforming growth factor-beta (TGF-β) [10,11]. These cytokines may promote vascular hyperpermeability, retinal inflammation, and disruption of retinal homeostasis, thereby increasing susceptibility to postoperative CME. More recently, soluble CD163 (sCD163), a marker of macrophage activation, has emerged as a potentially relevant biomarker reflecting inflammatory activity and disease severity in ERM, further supporting the hypothesis that inflammation may influence postoperative anatomical and functional recovery [12].

PPV with ERM peeling may further amplify this inflammatory cascade through surgical manipulation, retinal stress, and transient disruption of the blood–retinal barrier [7,8]. The resulting increase in inflammatory cytokines, combined with Müller cell dysfunction and vascular instability, may contribute to postoperative CME development, delayed anatomical recovery, and suboptimal visual recovery [8,13,14].

This inflammatory framework provides a plausible biological rationale for adjunctive DEX implantation during or after ERM surgery. By suppressing inflammatory cytokine release, stabilizing the blood–retinal barrier, and reducing retinal edema, DEX may help mitigate postoperative inflammation and improve anatomical recovery, particularly in selected high-risk patients [15,16].

## 5. Postoperative CME After ERM Surgery: Incidence and Risk Factors

Postoperative CME incidence ranges from 10% to 40% [15,16]. CME typically develops in the early postoperative period, although persistent forms may significantly affect long-term outcomes [3].

Its pathogenesis is multifactorial, in the context of pre-existing retinal structural alterations. Ahn et al. suggested that macular thickening from macular edema may be secondary to disruption of the blood–retinal barrier [7,16].

Govetto et al. identified microcystic macular changes as an OCT biomarker associated with inner retinal disruption and Müller cell dysfunction [8]. The anatomical continuity between Müller cells, ILM, and ERM may further compromise intraretinal fluid homeostasis, likely mediated by aquaporin water channels located at the ILM, and may also increase Müller cell susceptibility to surgical stress. The anatomical distribution of microcystoid macular edema (MME), which consistently appears beneath areas of persistent ERM–ILM adhesion, seems to support this hypothesis. During surgical maneuvers, regions with strong ERM–ILM adherence tend to behave as a single anatomical unit, as the ERM–ILM complex is often removed en bloc. This may increase the risk of iatrogenic Müller cell damage and impair the retina’s ability to clear interstitial fluid [13,14].

Consistently, MME has been linked to more advanced ERM stages, worse baseline visual acuity, and poorer postoperative outcomes, including a higher risk of persistent edema [9]. Moreover, perioperative factors contribute significantly to CME development.

Taken together, the available evidence suggests that postoperative CME after ERM surgery is driven by a combination of pre-existing retinal structural vulnerability, surgical trauma, inflammatory activation, Müller cell dysfunction, and blood–retinal barrier disruption. This mechanistic framework provides the biological rationale for selective adjunctive DEX use, particularly in patients at higher risk of postoperative inflammation and CME.

The extent of surgical manipulation, particularly during ERM and ILM peeling, intraoperative tractional stress, and adjunctive maneuvers such as dye-assisted visualization, may promote blood–retinal barrier breakdown and contribute significantly to CME development [8,15].

## 6. Evidence of Efficacy of Dexamethasone Implants in Epiretinal Membranes

Despite multiple studies investigating this question, the impact of dexamethasone implantation on functional outcomes remains incompletely defined. Efficacy is primarily assessed using functional and structural biomarkers that allow pre- and postoperative comparisons. These metrics are central to evaluating the therapeutic impact of DEX implantation. Currently, the biomarkers that are most commonly used are visual acuity, metamorphopsia and binocular function in terms of visual function and OCT parameters, such as central macular thickness and presence of intraretinal cysts. More recently with the advent of optical coherence tomography angiography (OCTA) vascular density studies have been used as an objective biomarker. The identification of such biomarkers allows a more precise assessment of the therapeutic impact of adjuvant treatments such as DEX implants. They also offer insights into the mechanisms of recovery and may help predict visual outcomes in patients undergoing ERM surgery, which in turn may help the surgeon to make customized treatment decisions [10,17]. For clinical interpretation, evidence should be considered separately according to both treatment timing (intraoperative/perioperative versus delayed DEX for persistent postoperative CME) and study design (prospective/randomized versus retrospective studies). Evidence regarding visual symptoms beyond BCVA, including metamorphopsia, binocular visual function, and patient-reported visual quality, remains limited. Most available studies focused primarily on BCVA and anatomical endpoints, with limited incorporation of symptom-based or patient-reported outcome measures.

### 6.1. Effects on Visual Acuity and Visual Symptoms

Best-Corrected Visual Acuity (BCVA) has been a standard metric in ophthalmology for decades. It reflects a crucial subjective outcome for patients and links anatomical improvements to functional vision. BCVA outcomes suggest that dexamethasone implantation provides more consistent anatomical benefit and earlier postoperative recovery than clear long-term functional superiority over PPV alone. As summarized in Table 1, evidence regarding the functional benefit of DEX implantation remains heterogeneous.

Direct quantitative comparison across studies remains challenging because functional outcomes were reported using heterogeneous visual acuity scales, follow-up intervals, and study designs, limiting meaningful cross-study aggregation of BCVA gains and clinically important differences. While several studies reported improved BCVA, particularly in eyes treated for persistent postoperative CME, others found no significant or sustained advantage over PPV alone.

Savastano et al. [11] reported an initially favorable response to DEX (improved at 15 days) that diminished over time (at 30 and 90 days), which aligns with the known bioavailability and pharmacokinetics of the drug. Guidi et al. [18] and Luan et al. [19], while demonstrating a trend to superiority of DEX over the control group, did not find a statistically significant difference between the groups. Similarly, Sane et al. [20] showed an initial improvement following DEX treatment (at 1 month), but failed to maintain statistical significance at 6 months post-surgery. In these studies, DEX was delivered perioperatively, either during surgery or in the early postoperative period.

There are relatively few studies in the literature that evaluate long-term functional recovery in terms of visual acuity, with some focusing specifically on the treatment of postoperative CME. In particular, Hattenbach et al. [21] demonstrated improvements in BCVA in patients treated with DEX for persistent CME following vitrectomy for ERM. In this study, intravitreal DEX was administered within 3 months of the primary surgery, but not in conjunction with it. The study reported a significant improvement within the first 65 days post-injection, with stability achieved by day 130. However, approximately 40% of patients required a second DEX injection within an average of 6.9 months. In the study conducted by Freissinger et al. [22], two groups of patients were analyzed: one group treated for ERM surgery, and another treated for retinal detachment, both receiving slow-release DEX implants for postoperative persistent CME. Although the study lacked a control group, the authors highlighted that lower baseline visual acuity and an intact EZ were potential predictors of a favorable response at 12 months following DEX implantation. Chang YC et al. [23] further demonstrated the efficacy of a single slow-release DEX injection for treating persistent CME following PPV for ERM. Patients in this study received DEX injections no earlier than 12 months after their initial PPV for idiopathic ERM and were subsequently divided into control and treated groups. The study reported significant advantage in the DEX group in terms of BCVA over a 12-month period. Overall, the available evidence suggests meaningful heterogeneity across studies evaluating dexamethasone (DEX) implantation in the setting of epiretinal membrane (ERM) surgery. Studies investigating intraoperative DEX implantation during pars plana vitrectomy generally reported more consistent anatomical benefits, including faster resolution of postoperative inflammation and cystoid macular edema, than clear long-term functional superiority over surgery alone. In contrast, studies evaluating delayed DEX administration for persistent postoperative cystoid macular edema primarily suggested benefit in selected eyes with chronic or refractory edema. Importantly, findings from prospective and randomized studies were broadly consistent with those from retrospective series in suggesting a more reproducible anatomical than functional benefit. Overall, earlier functional improvement observed at short-term follow-up did not consistently translate into superior long-term visual outcomes, highlighting the need for cautious interpretation of the available evidence. Importantly, patient-centered functional outcomes remain underreported in the current literature. Very few studies evaluated metamorphopsia, binocular visual function, or validated patient-reported outcome measures. As a result, the impact of DEX implantation on subjective visual quality and daily visual function remains largely unknown. Moreover, studies evaluating intraoperative or perioperative DEX implantation generally reported modest early functional improvements with substantial inter-study variability, whereas studies assessing delayed DEX treatment for persistent postoperative CME tended to report greater functional benefit in selected chronic edema cases.

**Table 1:** Structured summary of clinical studies evaluating dexamethasone implants in ERM surgery.

Study	Design	Eyes	DEX Timing	Functional Outcome	Anatomical Outcome	Safety	F/U
Baldascino et al., 2024 [24]	Prospective comparative	25/25	Intraoperative	No benefit	OCTA improved	NR	3 m
Boz et al., 2024 [25]	Prospective comparative	22/20	Delayed (post-op CME)	Improved	CST ↓, DRIL improved	IOP↑	12 m
Chang et al., 2018 [23]	Retrospective comparative	20/20	Delayed (persistent CME)	Improved	CST ↓	IOP↑	6 m
Chatziralli et al., 2019 [26]	Prospective comparative	15/12	Delayed (persistent edema)	Improved	CST ↓	IOP↑	12 m
Freissinger et al., 2020 [22]	Retrospective, no control	46	Delayed (persistent CME)	Improved	CST ↓	IOP↑	12 m
Furino et al., 2014 [27]	Retrospective comparative	8-Aug	Perioperative	Improved	CST ↓	No significant IOP ↑	7 m
Guidi et al., 2018 [18]	Prospective comparative	30/30	Perioperative	No benefit	No benefit	NR	6 m
Hattenbach et al., 2017 [21]	Retrospective, no control	37	Delayed (<3 months post-op)	Improved	CST ↓	NR	4 m
Hostovsky et al., 2020 [28]	Prospective, no control	12	Perioperative	Improved	CST ↓	NR	6 m
Iovino et al., 2019 [29]	Prospective comparative	20/20	Perioperative	Improved	CST ↓	NR	6 m
Li et al., 2023 [30]	Prospective comparative	41/41	Perioperative	No sustained benefit	No sustained benefit	NR	3 m
Luan et al., 2024 [19]	Prospective RCT	24/24	Intraoperative	No benefit	No benefit overall	NR	6 m
Nie et al., 2024 [31]	Retrospective comparative	21/20	Concurrent with PPV	Improved	CST ↓, DRIL improved	No significant IOP ↑	6 m
Sane et al., 2020 [20]	Retrospective comparative	15/15	Perioperative	No sustained benefit	No sustained benefit	NR	6 m
Savastano et al., 2021 [11]	Retrospective comparative	20/20	Perioperative	Early benefit only	CST ↓	NR	3 m

Abbreviations: CME, cystoid macular edema; CST, central subfield thickness; DEX, dexamethasone implant; DRIL, disorganization of retinal inner layers; ERM, epiretinal membrane; F/U, follow-up; IOP, intraocular pressure; NR, not reported; OCTA, optical coherence tomography angiography; PPV, pars plana vitrectomy; RCT, randomized controlled trial.

### 6.2. Effects on OCT-Based Anatomical Parameters

The advent of OCT technology in the 1990s provided quantifiable and objective parameters to evaluate functional treatment responses. In particular, central subfield thickness (CST) has shown a strong correlation with visual acuity improvement following anti-VEGF treatment for diabetic CME in large-scale studies [32,33]. It has been subsequently used as an outcome measure in assessing the effect of intravitreal DEX for persistent CME [32]. Most clinical studies comparing visual function in patients undergoing ERM surgery with or without intravitreal DEX have focused on the relationship between improvement in BCVA and CST [11,18,19,20,21,22,25,26,27,28,29,30,31]. Anatomical outcomes from the principal clinical studies are summarized in Table 1.

Similar to the outcome of visual acuity, not all studies reported an improvement in CST post-surgery, in particular, some clinical studies such as Sane et al. [20] and Li et al. [30] showed an initial improvement of CST after DEX treatment (at 1 month), which was not maintained at 6 months and 3 months after treatment, respectively.

With the continuing advancement in technology, additional OCT biomarkers are being identified that may allow more precise anatomical-functional correlations. Future studies using these biomarkers may identify specific subgroups of patients who may derive particular benefit from intravitreal DEX implants, offering the potential for improved outcomes. Indeed, Luan et al. [19] conducted a comprehensive qualitative OCT analysis, gathering information not only on CST but also on the presence of CME, MME, hyperreflective foci (HRF), disorganization of retinal inner layers (DRIL), and the continuity of the inner segment/outer segment (IS/OS) layer. This was a prospective, single-masked, randomized, controlled clinical trial that included 48 eyes, randomly assigned into a control group and a group that underwent intravitreal DEX implantation (combined with PPV). Their findings suggested that certain OCT biomarkers, such as CME [34], MME [35], and DRIL [36], may be associated with a reduction in BCVA following vitrectomy for idiopathic ERM. Regarding the presence of HRF on OCT, there remains ongoing debate about their clinical significance and pathogenesis. The most widely accepted theory suggests that these foci may represent focal activations of microglial cells triggered by an inflammatory response [37]. In line with this, Luan et al. [19] correlated the presence of HRF with elevated concentrations of sCD163 in the vitreous fluid, a marker associated with M2 macrophage activation [12]. Additionally, they observed a greater reduction in CST at one month postoperatively in patients with higher levels of sCD163 in the vitreous fluid. While this correlation was not supported by statistically strong results, it does suggest that there may be a greater response in patients with predominantly inflammatory patterns, and that these patterns could potentially be identified through the presence of HRF.

Boz et al. [25], in a prospective randomized controlled study, evaluated the structure of the retinal layers using OCT during the follow-up of patients treated with PPV for ERM who developed CME following the procedure. They were divided into a control group and a DEX group, and their analysis focused on the integrity of the ellipsoid zone (EZ), the presence of DRIL, and CST to identify potential biomarkers that could predict a more favorable anatomical and functional response in patients treated with DEX implant. This approach aimed to develop a predictive model for personalized treatment. Their findings demonstrated that DEX implantation led to significant improvements in DRIL for the treatment of CME following PPV and ERM removal. In particular, improvements in BCVA and a reduction in CST were observed during long-term follow-up (12 months), with a clear relationship between the DRIL grade, EZ integrity, and successful outcomes. However, patients with higher-grade DRIL consistently exhibited lower final visual acuity compared to those with lower-grade DRIL, despite the improvements. DRIL is typically associated with the disorganization of retinal layers following retinal insult, particularly affecting the microcirculation [38,39]. DRIL has also been linked to a negative impact on BCVA due to its disruption of the transmission of visual stimuli, which is often related to the degeneration of Müller cells [40]. Notably, Nie et al. [31] demonstrated that in patients treated for advanced iERM with concurrent intravitreal DEX implantation, there was a significant correlation between the CST reduction and the DRIL improvement over a 6-month postoperative period. Furthermore, the Authors also demonstrated that this improvement was more pronounced in the group receiving DEX compared to the group treated with PPV alone.

### 6.3. Effects on OCT Angiography-Based Parameters

The progression of ERM and its correlations with microvascular alterations have been extensively documented in the literature [41,42,43]. Specifically, parameters such as vessel density (VD) and perfusion density (PD) of the superficial and deep capillary plexuses (SCP and DCP, respectively) in the macular region (6.4 mm × 6.4 mm centered on the fovea) and at the foveal level (within the 1 mm central ring of the ETDRS grid) were evaluated through OCTA analysis [41,42,43]. With regard to the effects of DEX as an adjunct to PPV in ERM surgery, Baldascino et al. [24] assessed the microvascular response by prospectively analyzing [25] eyes undergoing PPV with DEX implant and 25 eyes undergoing PPV alone. Notably, they found a significant increase in SCP (from 42.1 ± 4.1 to 45.6 ± 4.3%, *p* = 0.01) and DCP (from 38.5 ± 7.5 to 41.7 ± 4.2%, *p* = 0.03) VD in the first group only. Moreover, the authors also reported that the greatest postoperative improvement was observed in patients with more advanced stages of ERM (i.e., stage 4 according to Govetto et al. [8], particularly in the PD of both the SCP (from 40.8 ± 4.3 to 44.7 ± 5.3, *p* = 0.003) and DCP (from 39.0 ± 8.9 to 43.7 ± 5.4, *p* = 0.01). However, postoperative PD values remained consistently below those found in stage 3 ERMs. These findings suggest that DEX may provide benefits beyond inflammation control, potentially contributing to faster microvascular stabilization and improved anatomical recovery in selected patients. Taken together, these imaging biomarkers may contribute to risk stratification and could eventually support individualized treatment selection. However, their role in guiding DEX use remains exploratory, as none has yet been prospectively validated as a treatment-selection tool. A summary of the principal OCT and OCTA biomarkers relevant to visual outcomes and DEX treatment selection is presented in Table 2.

**Table 2:** Key OCT and OCTA biomarkers associated with visual outcomes and their potential implications for DEX treatment in ERM surgery.

Biomarker	Association with BCVA	Clinical Significance	Potential Role in Future DEX Treatment Selection
Central subfield thickness (CST) [11,19,25]	Increased CST generally associated with worse BCVA	Reflects retinal edema and anatomical distortion	May identify eyes at risk of postoperative CME
Cystoid macular edema (CME) [22,23,25]	Associated with reduced BCVA	Indicates active edema and retinal dysfunction	Potential predictor of favorable DEX response
Microcystic macular edema (MME) [8,19]	Associated with worse baseline and postoperative BCVA	Suggests Müller cell dysfunction and retinal stress	May identify high-risk inflammatory phenotype
Disorganization of retinal inner layers (DRIL) [25,31]	Strongly associated with worse BCVA	Reflects inner retinal disorganization	Potential biomarker of DEX responsiveness
Ellipsoid zone (EZ) disruption [22,25]	Associated with poor visual prognosis	Indicates photoreceptor damage	May predict limited functional recovery despite anatomical improvement
Hyperreflective foci (HRF) [12,19,37,44]	Associated with inflammation and worse BCVA	Marker of retinal inflammatory activity	May help identify eyes likely to benefit from DEX
OCTA vascular alterations [24]	Reduced vascular density associated with worse visual outcomes	Reflects microvascular dysfunction	Potential emerging biomarker for treatment stratification

From a practical perspective, biomarkers may support risk stratification and selective DEX use in ERM surgery. Structural biomarkers with stronger clinical support, including baseline CME, increased CST, DRIL severity, and ellipsoid zone disruption, may help identify eyes at higher risk of postoperative edema and suboptimal recovery. In contrast, biomarkers such as hyperreflective foci, OCTA vascular alterations, and inflammatory markers including sCD163 remain promising but largely exploratory and should currently be considered hypothesis-generating rather than definitive treatment-selection tools.

### 6.4. Critical Appraisal of Current Evidence

Despite the growing interest in DEX implantation as an adjunct to pars plana vitrectomy with epiretinal membrane peeling, the overall quality of the available evidence remains limited. The current literature is highly heterogeneous with respect to study design, sample size, timing of DEX administration, surgical techniques, outcome measures, and follow-up duration.

Most available studies are retrospective and involve relatively small cohorts, limiting statistical power and increasing susceptibility to selection bias and confounding. In addition, substantial variability exists regarding the timing of DEX administration, with some studies evaluating intraoperative implantation and others assessing delayed treatment for persistent postoperative cystoid macular edema. These populations likely represent distinct clinical scenarios and should be interpreted separately.

Prospective comparative studies and randomized controlled trials provide more robust evidence and generally support the conclusion that DEX implantation may accelerate anatomical recovery and reduce postoperative CME, particularly in the early postoperative period. However, even among higher-quality studies, consistent long-term superiority over PPV alone in terms of visual outcomes has not been clearly demonstrated.

Therefore, while the current evidence suggests a potential role for DEX in selected patients, the available literature remains largely hypothesis-generating and does not yet support definitive practice-changing recommendations.

## 7. Safety Profile of Dexamethasone Implants in ERM Surgery

Most of the literature on the safety of DEX implants highlights the potential side effects on both the anterior and posterior segments of the eye, the elevation of intraocular pressure (IOP) and the increased risk of cataract occurrence [9,45]. Careful monitoring of safety parameters remains important for understanding the risk–benefit profile of dexamethasone implantation in ERM surgery. In clinical practice, monitoring should include baseline assessment followed by postoperative intraocular pressure evaluation during the early postoperative period, particularly within the first weeks after DEX implantation, when steroid-induced pressure elevation is most likely to occur. From a practical standpoint, DEX implantation may offer the greatest benefit in patients with advanced ERMs, inflammatory imaging biomarkers, or increased risk of postoperative CME, whereas greater caution is warranted in eyes with glaucoma, ocular hypertension, high baseline IOP, known steroid responsiveness, or repeated steroid exposure. A practical monitoring approach includes IOP assessment at baseline, within 1–2 weeks, at 6 weeks, and at approximately 3 months after implantation.

### 7.1. Effects on the Intraocular Pressure

DEX carries the potentially significant side effect of elevating IOP, especially in susceptible individuals, because of the effects exerted on the trabecular meshwork where it is thought to lead to decreased aqueous humor outflow through Schlemm’s canal. This can result in Steroid-Induced Ocular Hypertension (SIOH), defined as an IOP of ≥25 mmHg post-injection or an IOP increase of ≥10 mmHg over the baseline values [46,47]. If left untreated, this rise in IOP can lead to secondary glaucoma and optic nerve damage, posing a serious risk to vision [46].

A summary of the safety data that has been reported in the literature is collated in Table 3. Two authors did not find any significant IOP changes during their follow-ups [27,31], whereas all the other studies analyzed converged in reporting a significant IOP rise following DEX implant [9,22,23,25,26,27,31]. However, it is important to note that in all cases the pressure could be well-controlled by using topical IOP-lowering drops only.

Several risk factors are linked to SIOH, including young age (under 6 years old), primary open-angle glaucoma (POAG), family history of POAG, type 1 diabetes mellitus, previous steroid-induced IOP elevation, connective tissue diseases, history of penetrating keratoplasty (PKP), high baseline IOP, and high myopia [45,48]. In particular, Malclés et al. reported a significant IOP elevation within the first 2 weeks post-injection among the steroid responders and patients with suspicious glaucomatous changes, which was successfully managed with topical antiglaucoma drops only [49]. Both short- and long-term DEX administration have been associated with SIOH, although long-term use carries a greater risk of developing ocular hypertension 48. Furthermore, the likelihood of IOP elevation was described to increase with the number of injections [45]. Moreover, with regard to the lens status, it was reported that the IOP elevation risk was comparable between phakic and pseudophakic eyes post-DEX injection (20.0% versus 19.7%, respectively, *p* = 0.9) [9].

### 7.2. Effects on the Anterior Segment

Over the past ten years, case reports have increasingly highlighted instances of DEX implants dislocated into the anterior chamber (AC) [25,50,51,52,53,54,55,56,57].

Recognized risk factors for DEX implant migration are the presence of iridotomy, prior PPV, previous complicated intraocular lens (IOL) implantation, pseudophakia or aphakia, iris reconstruction, zonular dehiscence and defective lens capsule [51]. These conditions can compromise the normal anatomical barriers in the eye, increasing the likelihood of the implant shifting into the anterior chamber [50]. A recent multicenter retrospective review has estimated the risk of migration at 1.6%, with a higher rate of implant migration in vitrectomized eyes (4.8%) [52]. Several large-scale studies have shown that the prevalence of DEX implant migration falls between 0.63% and 1.60% [50,51,52]. The average time between DEX implant injection and the detection of implant migration into the anterior chamber was reported to be 13 days, with a range of 5–44 days [50].

Although this complication is relatively rare, it has drawn significant attention due to its potential to cause severe damage to the corneal endothelium, leading to corneal edema, the most commonly related condition [47,54]. The risk of corneal edema is higher in patients with low endothelial counts and early migration of the implant (<3 weeks) [23]. Moreover, the incidence of clinically significant cataract was 21.73% in the study conducted by Ayaz et al. [55]. In the 12-month GENEVA study, which included 1256 eyes treated with two doses of DEX implant, 29.8% of patients experienced cataract progression [56]. In the study conducted by Mathis et al., cataract surgery was performed in 63 phakic eyes (39.9%) during the study period; the majority of these were after the first DEX injection [9]. Rarely, DEX implants can also be accidentally injected into the lens, leading to cataract formation [57].

### 7.3. Effects on the Posterior Segment

The potential adverse effects of DEX implant on the posterior segment were evaluated in several published studies [58,59,60,61,62,63,64,65].

In particular, a study documenting 3925 intravitreal DEX implants reported an incidence of endophthalmitis of 0.102%. All cases occurred within 1 week of the DEX implant, with an average onset of symptoms at 3.6 days (SD 1.64, range 1–5 days). On average, patients received 11.3 DEX implants (range 2–30 injections) before developing endophthalmitis [58]. Similarly, the MEAD study reported 1 case of endophthalmitis out of 1427 injections [59] while the GENEVA study reported no cases out of 1418 intravitreal DEX injections [56].

Two case reports have also described higher risk of acute retinal necrosis following DEX implants, particularly in immunocompromised individuals [60,61]. One study reported a 68-year-old immunosuppressed man developing the condition 74 days after receiving the injection, with serological tests confirming an active cytomegalovirus (CMV) infection [60]. Another case involved a 45-year-old immunocompetent woman with a history of encephalitis, who developed acute retinal necrosis after receiving the injection. Herpes simplex virus (HSV) was detected in her aqueous and vitreous humor via quantitative PCR [61].

In rare instances associated with vitreomacular traction, the intravitreal DEX injection has been reported to result in the formation of a macular pseudohole [62]. Moreover, evidence suggests that the number of intravitreal injections could be directly related to an increased ERM incidence [63].

In a vitrectomized eye, the injection should be performed after the vitreous chamber has been filled with balanced saline solution (BSS). Injection in an air-filled chamber is not recommended, as the lack of resistance may cause the implant’s kinetic energy to damage the retina [64]. In patients with a history of vitrectomy, vitreous and retinal hemorrhage have been reported following an intravitreal DEX injection for CME due to the implant potentially striking the retina [65].

Although DEX implantation is generally well tolerated, rare but potentially vision-threatening complications should also be considered, including implant migration in aphakic eyes or in eyes with compromised posterior capsule integrity, endophthalmitis, retinal complications, and severe steroid-induced ocular hypertension. Importantly, much of the current safety evidence is extrapolated from broader DEX literature across retinal vascular diseases and uveitis rather than being derived exclusively from ERM-specific cohorts. Therefore, safety estimates in ERM surgery should be interpreted with caution, particularly for rare adverse events and higher-risk subgroups.

## 8. Clinical Implications and Patient Selection

One of the most clinically relevant unanswered questions is whether DEX implantation should be considered routinely during PPV with ERM peeling or reserved for selected high-risk patients. Based on current evidence, routine DEX use in all ERM surgeries is not supported, as consistent long-term functional superiority over PPV alone has not been demonstrated despite encouraging anatomical results [11,19,22].

Available evidence suggests that the greatest benefit from adjunctive DEX implantation may be observed in selected patients at increased risk of postoperative inflammatory complications, CME, or delayed anatomical recovery. In particular, eyes with advanced ERM stages, preoperative CME or MME, significant DRIL, HRF, EZ disruption, or other OCT features suggestive of a more inflammatory phenotype may represent a subgroup that could potentially derive greater benefit, although these proposed selection criteria remain hypothesis-generating and require prospective validation before routine clinical adoption [19,25,31].

Several imaging biomarkers have shown promise for risk stratification, although their role in guiding treatment selection remains exploratory and requires prospective validation. DRIL appears particularly promising, as it has been associated with worse baseline visual function and may reflect significant inner retinal disorganization and inflammatory stress. Boz et al. reported improvement in DRIL following DEX treatment in patients with postoperative CME, suggesting a possible structural biomarker of treatment response [25]. Similarly, Nie et al. demonstrated improved anatomical outcomes in patients with advanced idiopathic ERM receiving adjunctive DEX, further supporting the concept that biomarker-defined high-risk eyes may derive greater benefit [31]. Although hyperreflective foci, microcystic macular edema, and OCTA-derived vascular parameters remain more exploratory, they may help identify inflammatory phenotypes that could respond favorably to corticosteroid treatment [19,24].

Clinical factors should also be integrated into the decision-making process. Patients with diabetes mellitus, previous postoperative CME, chronic retinal inflammation, or other systemic and ocular risk factors for postoperative edema may be considered potential candidates for adjunctive DEX implantation. In contrast, eyes without preoperative edema, without biomarker-defined risk factors, and with limited inflammatory burden may derive only marginal benefit from routine steroid use.

Importantly, the potential anatomical benefits of DEX implantation must always be weighed against treatment-related risks, particularly intraocular pressure elevation. Therefore, caution is warranted in patients with glaucoma, ocular hypertension, known steroid responsiveness, or advanced optic nerve vulnerability, where the risks of corticosteroid exposure may outweigh potential benefits.

From a practical perspective, DEX implantation may currently be best viewed as a selective adjunctive strategy rather than a routine component of ERM surgery. A personalized risk–benefit approach integrating preoperative OCT biomarkers, systemic risk factors, and ocular comorbidities is likely the most rational strategy for optimizing patient selection. Future prospective studies should aim to validate biomarker-guided treatment algorithms and define more precise clinical scenarios in which DEX implantation provides meaningful functional and anatomical advantages. Overall, the risk–benefit profile of adjunctive DEX implantation appears most favorable in selected patients at higher risk of postoperative inflammation and CME, particularly those with advanced ERMs or biomarker-defined high-risk features. While DEX may accelerate anatomical recovery and improve inflammatory control, these potential benefits must be balanced against the risks of steroid-related ocular hypertension, cataract progression, implant migration, and rare vision-threatening complications. Careful patient selection and postoperative monitoring remain essential.

**Figure 1:**
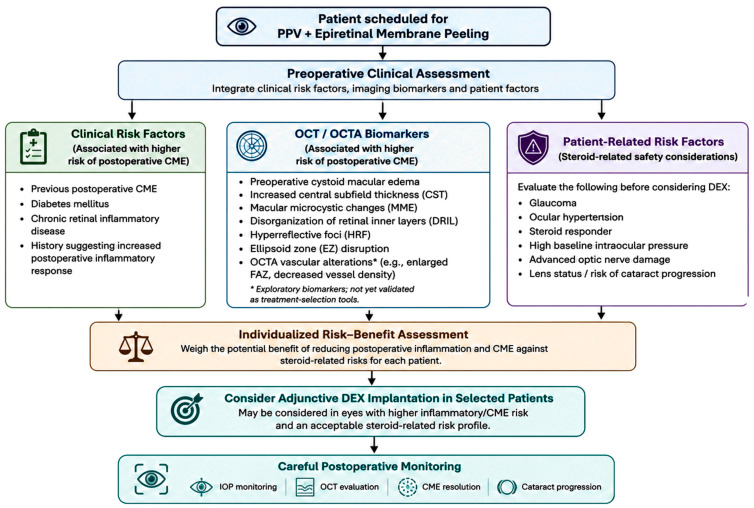
The framework integrates preoperative clinical characteristics, imaging biomarkers, and steroid-related safety considerations to support individualized risk–benefit assessment. It is intended solely as a conceptual summary of the currently available evidence and should not be interpreted as an evidence-based clinical algorithm. Prospective validation is required before biomarker-guided treatment selection can be implemented in routine clinical practice.

Based on the currently available evidence, Figure 1 presents a conceptual expert-opinion framework illustrating how clinical characteristics, imaging biomarkers, and steroid-related safety considerations may be integrated into individualized decision-making regarding adjunctive DEX implantation. This framework is intended solely to summarize the available evidence and identify potential candidates for future biomarker-guided treatment strategies; it does not represent an evidence-based clinical recommendation and requires prospective validation.

## 9. Limitations

A number of studies have explored the use of intravitreal dexamethasone implants in conjunction with pars plana vitrectomy for ERM surgery; however, the overall level of evidence remains limited. Many of the available reports are retrospective and include relatively small cohorts, which may reduce the strength and generalizability of the findings. In several instances, the data derive from uncontrolled case series, making it difficult to distinguish the specific contribution of the dexamethasone implant from the effect of the surgical procedure itself. In addition, there is considerable variability across studies with regard to the timing of DEX administration, as some authors used the implant intraoperatively while others employed it for the management of persistent postoperative CME. Several key limitations should be considered when interpreting the current evidence. First, most available studies are retrospective, non-randomized, and involve relatively small sample sizes, increasing susceptibility to selection bias and limiting internal validity.

Second, substantial heterogeneity exists across studies in terms of DEX timing, outcome definitions, follow-up duration, OCT/OCTA acquisition protocols, and concomitant surgical procedures, limiting direct comparability.

Third, important confounding factors—including cataract progression, lens status, combined cataract surgery, and prior intravitreal therapies—may have influenced both anatomical and functional outcomes.

Finally, reporting bias remains relevant, particularly given the limited availability of patient-reported outcomes, metamorphopsia data, and binocular visual function assessments, as well as the restriction to English-language literature.

Taken together, these limitations indicate that the current evidence base remains primarily hypothesis-generating and does not support definitive practice-changing recommendations. At present, adjunctive DEX use should be considered selectively and individualized according to patient risk profile, imaging biomarkers, and clinical context.

## 10. Conclusions and Future Perspectives

Current evidence suggests that dexamethasone (DEX) implantation may represent a useful adjunctive strategy in selected patients undergoing pars plana vitrectomy with epiretinal membrane (ERM) peeling, particularly in eyes at increased risk of postoperative inflammation, cystoid macular edema (CME), or delayed anatomical recovery.

The available literature more consistently supports a potential role for DEX in accelerating anatomical recovery and improving postoperative OCT-based parameters, particularly central subfield thickness and CME resolution. However, evidence regarding superior long-term functional outcomes, including visual acuity improvement, remains inconsistent across studies.

Importantly, the current evidence base is limited by substantial heterogeneity, small sample sizes, and the predominance of retrospective studies. Therefore, routine DEX implantation in all ERM surgeries cannot currently be recommended.

Future research should move beyond evaluating overall treatment efficacy and instead focus on identifying which patients are most likely to benefit from DEX implantation. Biomarker-guided patient selection using OCT and OCTA parameters—including CME, microcystic macular edema, disorganization of retinal inner layers, hyperreflective foci, ellipsoid zone integrity, and microvascular alterations—may represent a promising strategy for personalized treatment selection.

Additional key areas for future investigation include the optimal timing of DEX administration, the role of early versus delayed treatment, and the potential benefit of repeated DEX implantation in eyes with persistent or recurrent postoperative CME. Future prospective studies should aim to validate biomarker-guided treatment algorithms through adequately powered randomized controlled trials with standardized inclusion criteria, harmonized OCT/OCTA assessment, clearly defined functional and anatomical endpoints, and longer follow-up.

## Figures and Tables

**Table 3:** Intraocular pressure elevation after dexamethasone implantation in eyes treated for ERM-related cystoid macular edema.

References	IOP Elevation (>25 mmHg or >10 mmHg from Baseline)	F-UP	Eyes Receiving Intraoperative DEX with PPV	Eyes Receiving Delayed DEX for Postoperative CME
Boz et al. [25]	Yes	12 MO	0	22
Chang et al. [23]	Yes	12 MO	0	20
Chatziralli et al. [26]	Yes	12 MO	0	27
Freissinger et al. [22]	Yes	12 MO	0	61
Furino et al. [27]	No	6 MO	0	8
Mathis et al. [9]	Yes	19 MO	0	328
Nie et al. [31]	No	6 MO	20	21

CME: cystoid macular edema, DEX: dexamethasone, F-UP: follow-up, IOP: intraocular pressure, MO: months, PPV: pars plana vitrectomy.

## Data Availability

No new data were created or analyzed in this study. Data sharing is not applicable to this article.
